# Research and application of multi-component sterol determination methods in pre-prepared dishes

**DOI:** 10.3389/fnut.2025.1657372

**Published:** 2025-10-21

**Authors:** Ying Ying, Zhengyan Hu, Pinggu Wu, Xianfa Luo, Liyuan Wang, Jing Chen

**Affiliations:** ^1^Zhejiang Provincial Center for Disease Control and Prevention, Hangzhou, China; ^2^NHC Specialty Laboratory of Food Safety Risk Assessment and Standard Development, Hangzhou, China; ^3^Hangzhou Medical College, Hangzhou, China

**Keywords:** sterols, gas chromatography–mass spectrometry, pre-prepared dishes, saponification, derivatize

## Abstract

**Background:**

Pre-prepared dishes contain fats/oils, high protein, and complex seasonings, making sterol detection difficult due to multiple components and matrix interference. Given the market’s analytical challenges, detecting sterols—key functional components affecting nutritional value—is practically vital. This study aims to develop a sensitive, selective GC–MS method for simultaneous qualitative and quantitative multi-component sterol analysis in pre-prepared dishes and to examine their compositional traits.

**Methods:**

After saponification treatment, the sample undergoes ultrapure water-assisted dispersion and n-hexane extraction. The extract is dried and subjected to derivatization reaction. The derivative is redissolved and analyzed by gas chromatography–mass spectrometry (GC–MS) for qualitative identification, with quantification performed using the internal standard method. This method optimizes sample pretreatment and chromatographic separation conditions, enhancing detection efficiency and separation effectiveness.

**Results:**

The six target sterol compounds exhibited good linearity within the concentration range of 1.0–100.0 μg/mL (correlation coefficients ≥0.99). The limits of detection (LODs) and limits of quantification (LOQs) were 0.05–5.0 mg/100 g and 0.165–16.5 mg/100 g, respectively. At low, medium, and high spiked concentrations, the average recoveries ranged from 87.0 to 106%, with relative standard deviations (RSDs, *n* = 6) of 0.99–9.00%. Application of this method to analyze actual pre-prepared dish samples revealed significant variations in cholesterol content among different dish categories, with meat ingredients playing a dominant role. The sterol composition exhibited marked diversity: ergosterol was not detected in pre-prepared dishes, while β-sitosterol, campesterol, and stigmasterol constituted the major components. Notable differences in sterol content and composition were observed across different categories of pre-prepared dishes, further confirming the impact of various meat raw materials and processing technologies on sterol levels.

**Conclusion:**

The GC–MS analytical method established in this study has been validated to demonstrate excellent reliability and applicability, providing an efficient analytical tool for precise detection of multi-component sterols in pre-prepared dishes. This method supports quality control and nutritional value assessment in the pre-prepared dish industry, facilitating product labeling standardization and informed consumer choices.

## 1 Introduction

With the rapid expansion of the pre-prepared dish consumption market, serving as a significant source of dietary sterol intake, precise determination of its sterol composition and content is crucial for nutritional evaluation and safety control (1–3). However, processing techniques such as high-temperature thermal processing and repeated oil use in pre-prepared dish manufacturing tend to induce oxidative degradation and structural isomerization of sterols (4–6). These changes not only alter their nutritional functions but also introduce technical challenges such as isomer interference and reduced target identification for existing analytical methods, necessitating tailored analytical solutions. Therefore, establishing a detection method capable of accurately tracking sterol lineage changes during processing holds urgent practical significance for achieving the dual objectives of “safety-nutrition” assurance in pre-prepared dishes.

Current research on sterol detection primarily focuses on matrices such as vegetable oils (7), edible fungi (8), and Baijiu (9). However, the complex system formed by the “coexistence of animal and plant raw materials” in pre-prepared dishes poses significant challenges to existing methods: Gas chromatography (GC) lacks sufficient specificity (10, 11), making it difficult to distinguish sterol isomers generated during processing. While liquid chromatography–mass spectrometry (LC–MS) exhibits strong anti-interference capability (12, 13), eliminates the need for derivatization, and offers high sensitivity and specificity (14), its high instrument costs, complex maintenance requirements, and suboptimal separation efficiency for certain sterols (15) limit its suitability for batch testing. The vegetable oil sterol detection standard (GC–MS method) recommended by China’s Ministry of Agriculture, though serving as an industry benchmark, involves procedures such as heating reflux saponification, separatory funnel extraction, solid-phase extraction, and derivatization (7, 16). When applied to pre-prepared dishes, these steps not only suffer from severe matrix interference but also require lengthy pretreatment times and excessive solvent consumption, rendering them unsuitable for high-throughput analysis.

Notably, as pre-prepared dishes become an increasingly important source of sterol intake, their matrix complexity and processing-induced sterol morphological changes make existing methods difficult to balance detection accuracy and efficiency. Addressing this core contradiction, this study focuses on the matrix characteristics of pre-prepared dishes, establishing an efficient GC–MS analytical protocol for multi-component sterols by optimizing sample pretreatment and detection processes. This protocol aims to resolve the shortcomings of traditional methods in complex matrices—including insufficient specificity, cumbersome operation, and batch processing difficulties—by improving pretreatment efficiency. Specifically, liquid–liquid extraction using centrifuge tubes replaces traditional large-volume separatory funnel extraction, while constant-temperature oscillating water bath saponification substitutes heating reflux, reducing organic solvent usage. This provides technical support for precise sterol detection in pre-prepared dishes, thereby serving industrial quality control and residential dietary assessment.

## 2 Materials and methods

### 2.1 Instruments and reagents

7890B/5977A Gas Chromatography–Mass Spectrometry (GC–MS) (Agilent Technologies, USA); Analytical Balances [readability: 0.1 mg, Mettler Toledo Instruments (Shanghai) Co., Ltd.]; SW22 Thermostatic Shaking Water Bath (Julabo GmbH, Germany); Multi-Function Vortex Mixer (Heidolph Instruments GmbH & Co. KG, Germany); EZ-2 Vacuum Centrifugal Concentrator (GeneVac Ltd., UK); Water Bath Nitrogen Evaporator [ANPEL Laboratory Technologies (Shanghai) Inc., China].

Stigmasterol, β-sitosterol, and ergosterol were purchased from Shanghai ANPEL Experimental Technology Co., Ltd. (Shanghai, China), All with purity ≥99.5%; campesterol, brassicasterol, and cholestane (internal standard) were obtained from Tanmo Technologies Co., Ltd. (Changzhou, China), All with purity ≥99.5%; cholesterol was supplied by Yuanye Bio-Technology Co., Ltd. (Shanghai, China), purity ≥99.0%. N,O-bis(trimethylsilyl)trifluoroacetamide (BSTFA) containing 1% trimethylchlorosilane (TMCS) (v/v = 99:1), HPLC-grade n-hexane, and absolute ethanol were sourced from Fisher Scientific (USA). Potassium hydroxide (AR grade) was provided by Sinopharm Chemical Reagent Co., Ltd. (China).

### 2.2 Chromatography–mass spectrometry conditions

#### 2.2.1 Chromatography conditions

DB-5MS Capillary Column (30 m × 250 μm × 0.25 μm, 5%-phenyl-methylpolysiloxane); Temperature Program: Initial 100 °C (1 min hold), ramp at 20 °C/min to 220 °C, then at 5 °C/min to 270 °C (5 min hold), followed by 2 °C/min to 290 °C (5 min final hold), Total run time 37 min; Injector Temperature: 290 °C; Carrier Gas: High-purity helium, 99.999%; Flow Rate: 1.0 mL/min; Split Ratio: 10:1; Injection Volume: 1.0 μL.

#### 2.2.2 Mass spectrometry conditions

Electron Ionization (EI) Source; Ion Source Temperature: 230 °C; Quadrupole Temperature: 150 °C; Interface Temperature: 280 °C; Electron Energy: 70 eV; Solvent Delay: 10 min; Acquisition Mode: Selected Ion Monitoring (SIM).

### 2.3 Preparation of solutions

Accurately weigh individually approximately 10 mg (accurate to 0.1 mg) of cholesterol, brassicasterol, ergosterol, campesterol, stigmasterol, and β-sitosterol reference standards individually into 10 mL glass volumetric flasks. Each standard was dissolved in n-hexane and diluted to volume to prepare 1.0 mg/mL sterol standard stock solutions, which were stored below 4 °C, protected from light, with a shelf life of 6 months. Working standard solutions at varying concentrations were prepared by serial dilution with n-hexane immediately before use. For internal standard solution, accurately weigh approximately 10 mg (accurate to 0.1 mg) of cholestane into a 10 mL glass volumetric flask, dissolved in n-hexane, and diluted to volume to obtain a 1 mg/mL internal standard solution, which was stored below 4 °C, protected from light, with a shelf life of 6 months.

### 2.4 Preparation of standard curve

Accurately pipette 0.01 mL, 0.02 mL, 0.04 mL, 0.10 mL, 0.50 mL, and 1.0 mL of 1.0 mg/mL sterol standard stock solution into individual 10 mL glass volumetric flasks. Add 0.50 mL of 1.0 mg/mL internal standard solution (cholestanol) to each flask, then dilute to volume with n-hexane to prepare a standard series with concentrations of 1.0 μg/mL, 2.0 μg/mL, 5.0 μg/mL, 10.0 μg/mL, 50.0 μg/mL, and 100.0 μg/mL. The internal standard concentration in all standard series solutions is maintained at 50 μg/mL. These standard solutions undergo derivatization simultaneously with the samples.

### 2.5 Sample preparation

The monitoring samples were sourced from various links such as supermarkets, farmers’ markets, and online shopping platforms. A total of 37 samples of prepared dishes were collected. The edible parts (including main ingredients, auxiliary ingredients, and seasonings) of each collected prepared dish sample were poured into a homogenizer (with bones, fish bones, etc., removed), ground up, and then transferred into two 50—mL plastic centrifuge tubes. The samples were stored frozen at −18 °C, with one tube used for testing and the other reserved for re-testing as a retained sample. A 2.0 g portion (accurate to 0.1 mg) was weighed into a 50 mL polypropylene centrifuge tube, followed by addition of 50 μL internal standard working solution and 15 mL absolute ethanol. The mixture was vortex-mixed, then supplemented with 5 mL 60% (w/w) potassium hydroxide solution with additional 1 min vortexing. Saponification was performed in a 75 °C thermostatic shaking water bath for 0.5 h to ensure complete reaction. After cooling to ambient temperature, 10 mL ultrapure water was added with vortex mixing. Subsequent liquid–liquid extraction involved sequential addition of 15 mL n-hexane (5 min vortex extraction) and 10 mL n-hexane (repeated extraction), with combined organic phases collected. The extraction solvent was washed to neutrality with 20–40 mL ultrapure water under gentle agitation to prevent emulsification. The organic layer was evaporated to dryness under vacuum centrifugal concentration, followed by 30 min drying at 85 °C. After cooling, the residue was reconstituted with 100 μL BSTFA silylating reagent, capped, and heated at 75 °C for 0.5 h for derivatization. The final solution was cooled, brought to 1.0 mL with n-hexane, and transferred for GC–MS analysis.

## 3 Results and discussion

### 3.1 Optimization of detection conditions

#### 3.1.1 Selection of internal standard

Consequently, sample pretreatment involving purification, enrichment, and concentration steps is essential. These procedures may induce target compound loss, compromising recovery efficiency. The internal standard method was therefore deemed necessary for accurate quantification. Through preliminary screening, cholestane was found absent in pre-prepared dish matrices while exhibiting similar physicochemical properties to target sterols. It demonstrated complete miscibility with sample matrices, distinct chromatographic separation from analyte peaks, and co-elution proximity without interference. This profile effectively mitigates sensitivity variations arising from instrumental instability. Cholestane was thus selected as the optimal internal standard for this analytical methodology.

#### 3.1.2 Selection of characteristic ions

Full scan mode was employed to analyze the 1 mg/mL mixed standard solution containing six sterol compounds. Characteristic ions exhibiting high abundance, minimal interference, and optimal spectral matching were selected as qualitative/quantitative markers for each analyte, with retention times tentatively established. Subsequent analysis utilized selected ion monitoring (SIM) mode to enhance method selectivity and sensitivity.

#### 3.1.3 Selection of injection conditions

**Figure 1:**
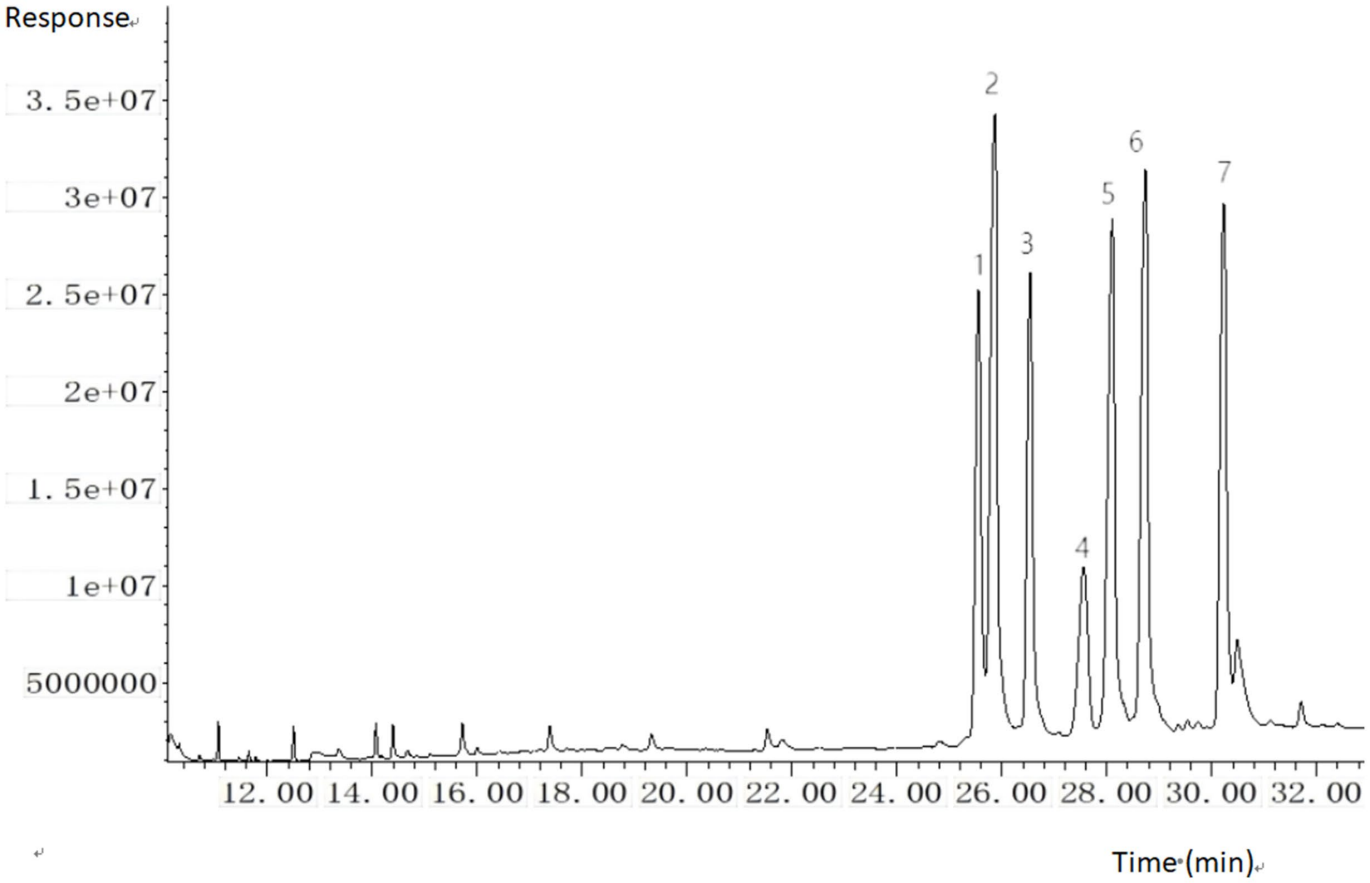
GC–MS total ion chromatogram (TIC) of six sterols and internal standard (trimethylsilyl derivatives). 1. cholesterol; 2. cholestane (IS); 3. brassicasterol; 4. ergosterol; 5. campesterol; 6. stigmasterol; 7. β-sitosterol.

The split ratio of 10:1 was adopted for injection, primarily based on the balanced optimization of sensitivity and peak shape. During preliminary experiments, comparisons of different split ratios (e.g., 5:1, 10:1, 20:1) revealed that at 10:1, the response signals of target analytes meet detection sensitivity requirements while effectively avoiding chromatographic peak broadening caused by excessively low split ratios. This ratio also reduces the risk of system contamination from high-concentration matrix introduction. The parameter demonstrated good stability and reproducibility in preliminary tests, and was therefore ultimately determined to ensure detection efficiency and data reliability. The trimethylsilyl (TMS) derivatives of six sterols and the internal standard were chromatographically resolved, with specific retention times and diagnostic ions detailed in Figure 1; Table 1.

**Table 1:** Quantitative ions, qualitative ions and retention time table of sterols (trimethylsilyl ethers).

Compound	Quantitative ion (m/z)	Qualitative ion (m/z)			Retention time (min)
Cholesterol	330.4	355.4	370.4	460.5	25.496
Cholestane	445.5	460.5	403.4	461.5	25.752
Brassicasterol	380.4	470.5	365.4	341.4	26.456
Ergosterol	363.4	337.3	378.4	468.5	27.459
Campesterol	382.4	343.4	367.4	472.5	27.992
Stigmasterol	394.4	484.5	379.4	469.5	28.632
β-sitosterol	396.4	357.4	381.4	486.5	30.147

### 3.2 Optimization of pretreatment conditions

#### 3.2.1 Optimization of saponification conditions

##### 3.2.1.1 Selection of saponification temperature

**Figure 2:**
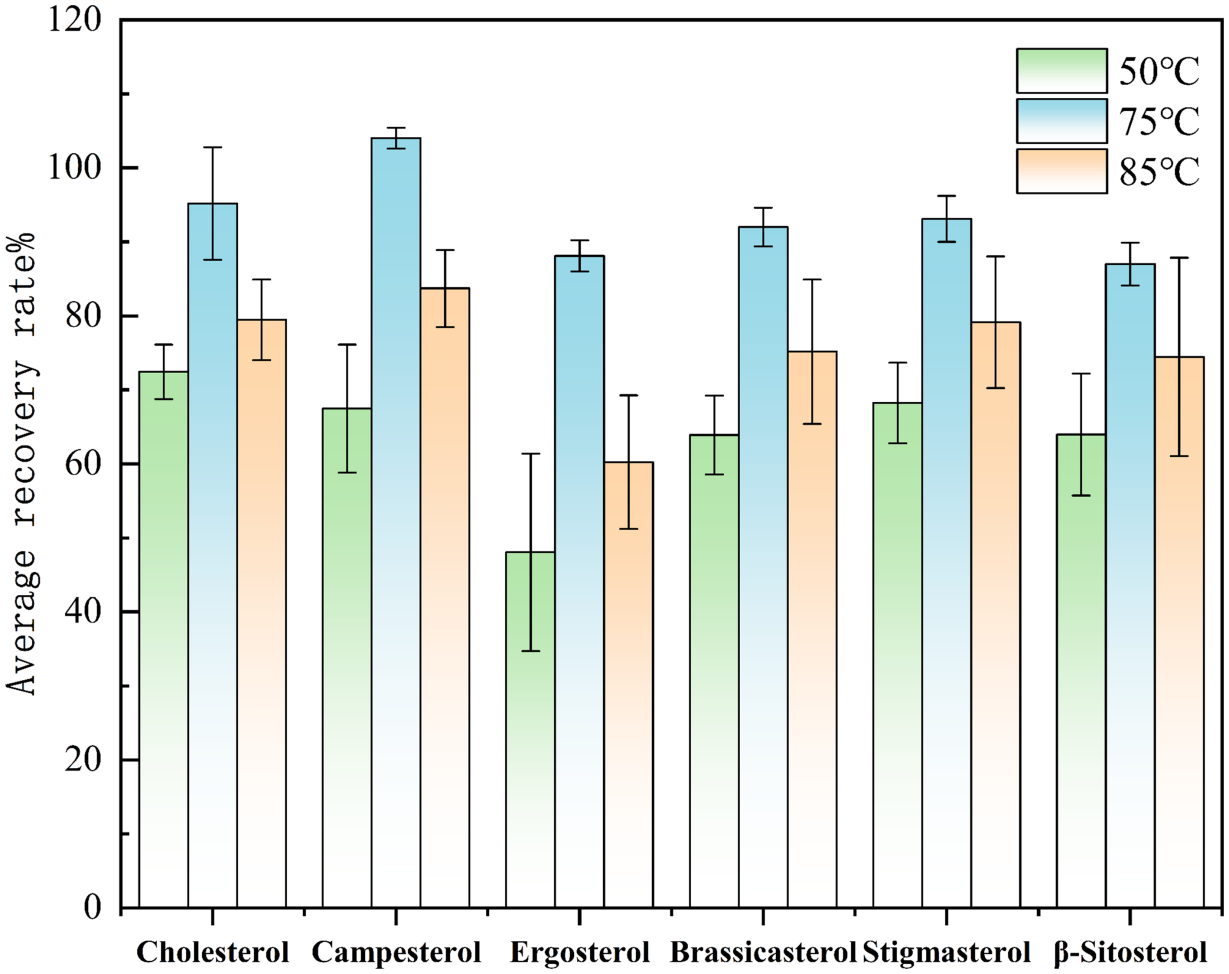
Comparison of the spiked recovery rates of sterols under different saponification temperatures.

Select Huangguo (a type of rice food) samples with a low background level. Add 0.01 mL of a sterol standard stock solution with a concentration of 1.0 mg/mL and 0.50 mL of an internal standard solution with a concentration of 1.0 mg/mL. Under the same other pretreatment and detection conditions, saponification was performed at temperatures of 50 °C, 75 °C, and 85 °C to investigate the effects of different temperatures on sterol saponification. Conduct each temperature test more than six times. Calculate the measured values using the internal standard method, and then calculate the recovery rates and relative deviations. Plot graphs with the average recovery rates and relative deviations of each sterol under different temperatures as the vertical coordinates. The experimental results showed that as the saponification temperature increased, the response value of sterol compounds gradually increased. However, when the temperature exceeded 75 °C, the spiked recovery rates decreased, which may be related to the thermal decomposition of sterols caused by high temperatures under alkaline conditions. Therefore, this study selected 75 °C as the saponification temperature, which meets the saponification conditions and is less prone to thermal decomposition reactions. The effect of saponification temperature on the spiked recovery rates of sterols is shown in Figure 2.

##### 3.2.1.2 Selection of saponification time

**Figure 3:**
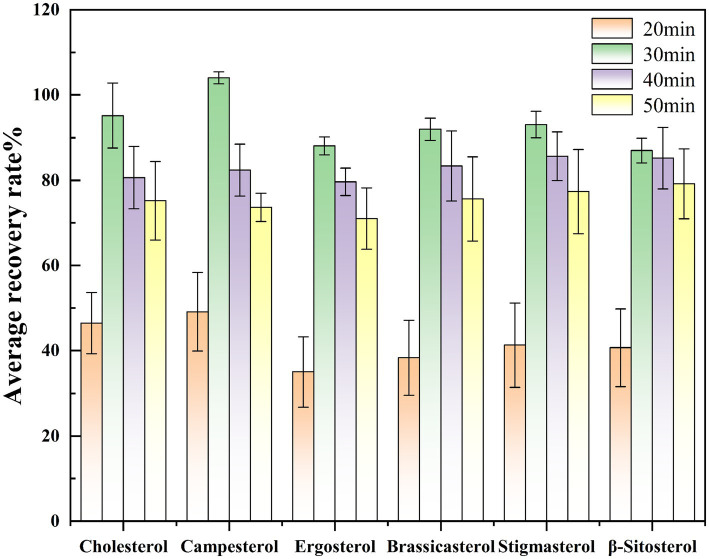
Comparison of sterol spiked recovery rates at different saponification times.

Experiment on the selection of the same saponification temperature Under the same pretreatment and detection conditions, with the saponification temperature maintained at 75 °C, saponification was conducted for 20, 30, 40, and 50 min to investigate the effect of saponification time on sterol saponification efficiency under consistent temperature conditions. Based on the spiked recovery experiment, the condition with the highest average recovery rate indicates the optimal saponification time. The experimental results indicated that the spiked recovery rates of sterol compounds were initially low at 20 min, gradually increased with prolonged saponification time, peaked at 30 min, and then declined thereafter. Therefore, 30 min was selected as the optimal saponification time for this study. The effect of saponification time on the spiked recovery rates of different compounds is illustrated in Figure 3.

#### 3.2.2 Selection of derivatization conditions

##### 3.2.2.1 Derivatization reagent selection

Due to the presence of polar hydroxyl groups in sterol structures and their inherently low volatility, detection via gas chromatography–mass spectrometry (GC–MS) necessitates derivatization treatment. Common derivatization methods include silylation, esterification, and acylation (17). Silylation is particularly suitable for compounds containing polar functional groups such as hydroxyl, amino, or carboxyl groups, as the resulting silylated derivatives exhibit improved volatility and thermal stability, rendering them more compatible with GC–MS analysis. Consequently, BSTFA [N,O-bis(trimethylsilyl)trifluoroacetamide] was selected as the derivatization reagent for this study.

##### 3.2.2.2 Selection of derivatization time and temperature

**Figure 4:**
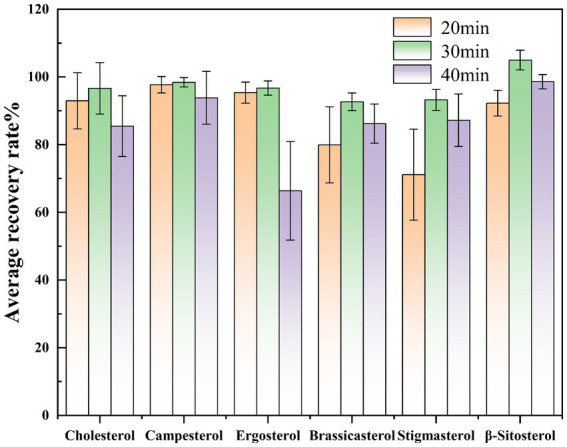
Comparison of spiked recovery rates of sterols at different derivatization times.

Based on existing literature (18), the effect of derivatization time on sterol determination was investigated by maintaining a constant derivatization temperature and varying the reaction duration (20 min, 30 min, and 40 min). We selected Huangguo (a type of rice food) samples with a low background level, added 0.01 mL of a 1.0 mg/mL sterol standard stock solution and 0.50 mL of a 1.0 mg/mL internal standard solution. For each time point, the experiment was repeated over six times. The measured values were calculated using the internal standard method, followed by determination of the recovery rates and relative deviations. Graphs were plotted with the average recovery rates and relative deviations of each sterol under different derivation times as the vertical coordinates. Results indicated that while a 20-min derivatization period generally met the requirements for sterol derivatization, the recovery rates of certain sterols (e.g., campesterol, stigmasterol) remained suboptimal. Extending the derivatization time to 30 min achieved recovery rates exceeding 90% for all sterol compounds, confirming complete derivatization. However, prolonging the reaction to 40 min led to reduced recovery rates for specific sterols (e.g., ergosterol), likely due to side reactions or degradation under prolonged heating. Consequently, 30 min was selected as the optimal derivatization time (Figure 4).

**Figure 5:**
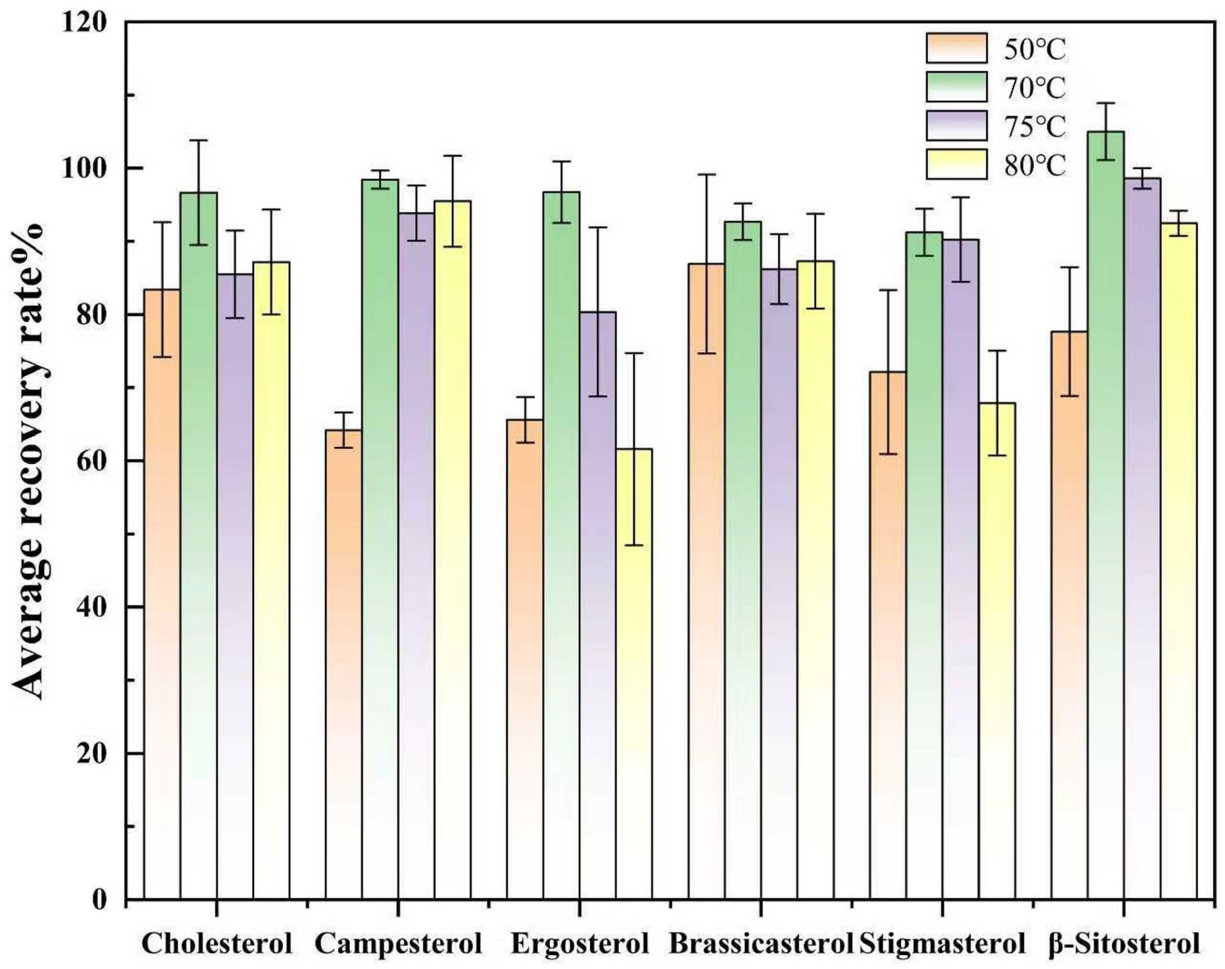
Comparison of spiked recovery rates of sterols at different derivatization temperatures.

Subsequently, the impact of derivatization temperature was evaluated by maintaining the optimized 30-min reaction time while varying the temperature (50 °C, 75 °C, 80 °C, 90 °C). Similarly, using the spiked recovery experiment. At 50 °C, incomplete derivatization was observed, as evidenced by lower recovery rates. Elevating the temperature to 75 °C resulted in complete derivatization, with all sterol recoveries exceeding 90%. Further temperature increases above 80 °C caused a decline in recovery rates, potentially due to hydrolysis or self-decomposition of the derivatization reagent, which reduced the effective reagent concentration and impaired derivatization efficiency (Figure 5).

### 3.3 Methodological validation

#### 3.3.1 Linear range, limit of detection (LOD), and limit of quantitation (LOQ)

Under the optimized analytical conditions, the linear range, LOD, and LOQ of the method were validated for six sterol compounds (trimethylsilyl ether derivatives). The results demonstrated excellent linearity across the concentration range of 1.0–100 μg/mL for all target sterols, with correlation coefficients (*r*^2^) exceeding 0.99. The LODs, calculated based on a signal-to-noise ratio (S/N) of 3, ranged from 0.005 mg/kg to 0.5 mg/kg. The LOQs, determined using an S/N of 10, ranged from 0.0165 mg/kg to 1.65 mg/kg. Detailed parameters, including linear equations, correlation coefficients, LODs, and LOQs for each sterol compound, are summarized in Table 2.

**Table 2:** Linear equations, correlation coefficients, limits of detection (LOD), and limits of quantitation (LOQ) for each sterol compound.

Sterol compounds (trimethylsilyl ether derivatives)	Linear range (μg/ml)	Regression equation	*r* ^2^	Detection limit (mg/kg)	Quantitative limit (mg/kg)
Cholesterol	1 ~ 100	*Y* = 1.461*x*−0.00033	0.998	0.005	0.0165
Campesterol		*Y* = 0.917*x*−0.00735	0.999	0.005	0.0165
Ergosterol		*Y* = 0.809*x*−0.03567	0.994	0.500	1.65
Brassicasterol		*Y* = 1.564*x*−0.02147	0.999	0.005	0.0165
Stigmasterol		*Y* = 1.764*x*−0.01794	0.999	0.005	0.0165
β-sitosterol		*Y* = 1.248*x*−0.01997	0.996	0.005	0.0165

#### 3.3.2 Accuracy and precision

To validate the method’s accuracy and precision, spike recovery experiments were conducted using qingke (hulless barley) samples with inherently low background levels of target sterols. Low, medium, and high concentration levels were spiked into the samples, followed by six replicate analyses at each concentration level. The results, summarized in Table 3, demonstrate that the average recoveries for the six sterol compounds ranged from 87.0 to 106%, with relative standard deviations between 0.99 and 9.00%. These outcomes confirm the method’s accuracy, reliability, and suitability for multicomponent sterol analysis in prepared dishes.

**Table 3:** Accuracy and precision of six sterol determinations in prepared dishes (*n* = 6, %).

Sterol compounds	Low concentration (1 μg/mL)		Medium concentration (10 μg/mL)		High concentration (50 μg/mL)	
	Average recovery rate %	Relative standard deviation %	Average recovery rate %	Relative standard deviation %	Average recovery rate %	Relative standard deviation %
Cholesterol	95.2	7.60	106	4.21	97.2	7.11
Campesterol	104	1.40	105	1.41	98.2	2.67
Ergosterol	88.1	2.10	94.2	9.00	103	2.40
Brassicasterol	92.0	2.61	92.6	1.80	91.0	2.03
Stigmasterol	93.1	3.11	93.8	1.29	90.9	2.11
β-sitosterol	87.0	2.91	93.0	0.99	95.2	8.70

### 3.4 Actual sample analysis

**Figure 6:**
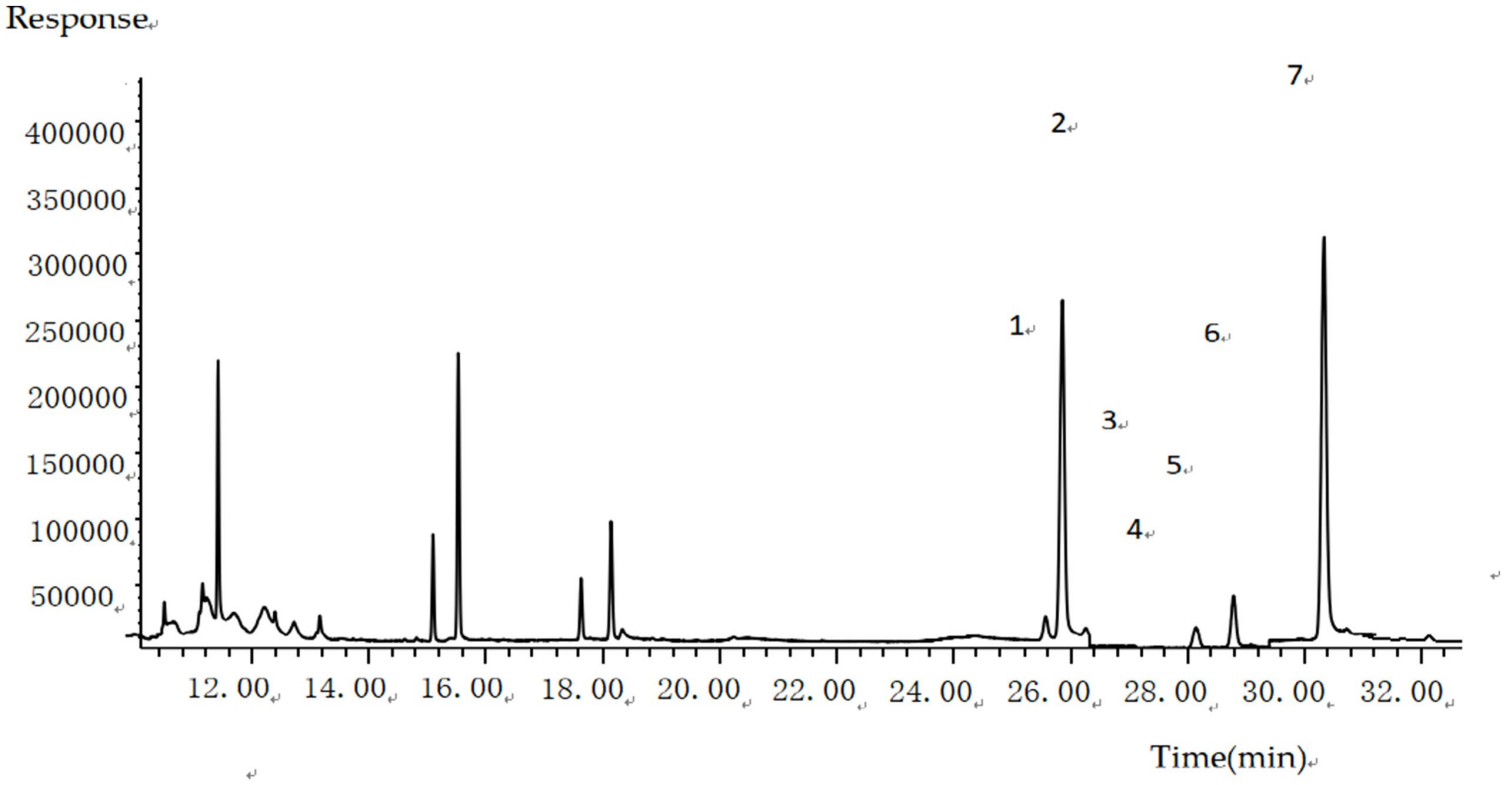
GC–MS total ion chromatogram of six sterols and an internal standard rimethylsilyl ether compound in boiled beef.

Using the method established in this study, we detected and analyzed cholesterol and various phytosterols (campesterol, ergosterol, campestanol, stigmasterol, and β-sitosterol) in 37 samples of finished dishes (including chicken, pork, beef, and other types of dishes). The results are shown in Table 4. The analysis revealed the significant influence of different meat ingredients and processing techniques on the sterol content. The research findings indicate significant differences in cholesterol content across various dish types, ranging from 0.1 mg/100 g in the purely vegetarian dish “Wufangzhai vacuum sweet lotus root” to 51 mg/100 g in the purely meat-based dish “Wufangzhai Dongpo pork,” reflecting the dominant role of meat ingredients in cholesterol levels. Regarding phytosterols, ergosterol levels were generally low and often undetectable in most samples. The detection of other phytosterols was closely associated with the addition of vegetable oil during the processing of prepared dishes. A further comparison of total sterol content across different meat-based dishes revealed that pork dishes generally had higher total sterol levels than chicken and beef dishes, ranging from 21.1 mg/100 g to 52.5 mg/100 g. This may be related to pork’s higher fat content and the promotion of sterol release in high-fat environments during processing (19). The total sterol content in chicken dishes was relatively low, ranging from 1.92 mg/100 g to 33.7 mg/100 g, which is attributed to their lower fat content and less retention of sterols during processing. Notably, the higher values within this range corresponded to fried products, indicating that cooking methods could potentially exert a greater influence on sterol levels than the raw ingredients themselves. In contrast, beef dishes exhibited a moderate total sterol content, ranging from 21.6 mg/100 g to 30.6 mg/100 g, approaching the lower end of the range observed in pork dishes. This can be attributed to the dense structure of beef muscle fibers and their weaker ability to adsorb oils. Detailed analysis of specific dishes, such as Daxidi vegetable chicken breast patties, Wufangzhai Dongpo pork, and Wufangzhai marinated beef, further corroborated the significant impact of different meat ingredients and processing techniques on sterol content (see Figure 6).

**Table 4:** Determination of sterol content in pre-prepared dishes.

Pre-prepared dishes	Cholesterol (mg/100 g)	Campesterol (mg/100 g)	Ergosterol (mg/100 g)	Brassicasterol (mg/100 g)	Stigmasterol (mg/100 g)	β-Sitosterol (mg/100 g)
Chicken dishes						
Daxidi vegetable chicken breast patty	11.2	0.0198	N. D.	1.76	0.384	10.2
Daxidi vegetable chicken breast patty	16.5	0.0188	N. D.	1.96	0.426	6.16
Daxidi dual-protein quinoa chicken cutlet	22.5	0.0213	N. D.	0.305	0.0444	0.52
Daxidi finger-lickin’ black pepper chicken bites	19.4	0.0388	N. D.	2.34	1.04	8.39
Daxidi orleans flavor chicken cutlet	16.8	0.0231	N. D.	0.413	0.176	1.49
Daxidi unforgettable chicken cutlet	22.9	0.0181	N. D.	0.23	0.0348	0.644
Daxidi vanilla chicken cutlet	31.9	0.0194	N. D.	0.259	0.0219	0.335
Daxidi pan-fried chicken thigh steak	29.3	0.0349	N. D.	0.968	0.41	3.03
Daxidi kids’ olive oil tender-fried chicken cutlet	29.6	0.0198	N. D.	0.276	0.0346	0.388
Maizima black pepper pork tripe and chicken stew	1.70	0.0194	N. D.	0.0517	0.027	0.121
Maizima black pepper pork tripe and chicken stew soup	2.00	0.0192	N. D.	0.0512	0.0268	0.0629
Wufangzhai lotus leaf wrapped beggar’s chicken	40.1	0.0261	2.34	0.689	0.237	1.71
Wufangzhai salt-baked chicken	8.80	0.105	N. D.	1.28	0.115	1.45
Wufangzhai salt-baked chicken	8.40	0.107	N. D.	0.943	0.126	1.48
Pork dishes						
Daxidi vegetable diet sausage	32.5	0.019	1.79	0.684	0.114	1.53
Maizima Taiwanese braised pork sauce	21.2	0.0324	7.70	2.39	1.15	8.72
Maizima juicy burst grilled sausage	19.6	0.0243	N. D.	0.751	0.100	0.906
Maizima Japanese-style tonkatsu	20.0	0.0678	N. D.	2.66	0.62	7.89
Maizima mini crispy pork	20.1	0.0673	N. D.	1.81	0.392	5.60
Maizima sweet and sour tenderloin	16.7	0.0202	N. D.	2.07	0.745	7.00
Maizima fish-fragrant shredded pork	12.9	0.0339	0.957	1.41	0.705	5.07
Maizima scallion pork	17.0	0.0847	N. D.	3.38	0.931	11.5
Maizima Guobaorou	18.9	0.0208	N. D.	1.61	0.636	5.80
Xiangtaifeng sweet and sour pork tenderloin	14.2	0.0369	N. D.	3.34	1.67	9.78
Xiangtaifeng sweet and sour pork ribs	17.6	0.0385	N. D.	4.44	2.29	5.67
Daxidi mini crispy pork	25.4	0.0338	N. D.	2.61	1.08	10.0
Wufangzhai Dongpo pork	51.5	0.0242	N. D.	0.607	0.0242	0.296
Beef dishes						
Daxidi kids’ beef steak	21.1	0.0215	N. D.	0.154	0.0347	0.305
Maizima poached beef in chili oil	16.0	0.0303	1.32	2.92	1.27	11.9
Maizima fresh sirloin steak with black pepper	28.7	0.0194	N. D.	0.514	0.257	1.85
Xiangtaifeng oyster sauce beef tenderloin	22.1	0.3676	N. D.	1.63	0.300	3.99
Wufangzhai marinated beef	28.4	0.0329	N. D.	1.36	0.0598	0.734
Other dishes						
Maizima lamb spine hot pot	23.6	0.0263	0.709	0.930	0.207	2.27
Daxidi deep sea cod fish cake	29.0	0.0406	N. D.	2.00	1.05	8.66
Maizima Laotan pickled cabbage fish	9.90	0.0409	N. D.	0.672	0.182	2.12
Wufangzhai vacuum-packed sweet lotus root	0.100	0.0186	N. D.	0.268	0.120	5.60
Huangguo	0.100	0.0192	N. D.	0.132	0.156	1.66

## 4 Conclusion

This study established a gas chromato (GC–MS) coupled with internal standard method for the determination of sterols in processed dishes. By integrating multidimensional methodological optimizations, this technique significantly enhances the resolution efficiency of sterol components in complex food matrices while retaining the intrinsic advantages of GC–MS. Key improvements include streamlined operational procedures, reduced analysis time, enhanced accuracy of results, and demonstrated high sensitivity and precision. By optimizing the coupling of pre-treatment procedures with chromatographic-mass spectrometric parameters, the analysis cycle was significantly shortened. Furthermore, the adoption of internal standard quantitative analysis effectively eliminated systematic errors introduced during sample preparation and instrumental fluctuations, thereby enhancing the method’s accuracy and precision. Finally, a mass spectrometry database targeting matrix interference in prepared dishes was constructed, enabling precise qualitative and quantitative analysis of multi-component sterols through characteristic ion pair screening and retention time locking techniques. This effectively resolves technical challenges in sterol detection for pre-prepared dishes, particularly interference from isomerization and significant matrix effects. This method not only provides reliable technical support for analyzing compositional differences in sterols across various meat products but also demonstrates significant application value in two dimensions: in-depth analysis of food nutritional components and precise quality control of prepared dishes. At the fundamental research level, it supports scientific exploration of dietary nutrient intake patterns; at the industrial application level, it offers critical quality control indicators for standardized production of prepared dishes. Compared to the GC–MS methodology developed by Zuo et al. (10) for determining multi-component sterols in camellia seed oil, where the method detection limits range from 0.22 mg/100 g ~ 2.30 mg/100 g, and the method by Du et al. (16) for determining phytosterols in edible oils using gas chromatography, with method detection limits of 3.00 mg/100 g ~ 11.00 mg /100 g, the present method demonstrates significant advantages in limit of detection (LOD) and substantially reduces experimental duration, making it more suitable for batch testing of pre-prepared dishes.

It should be noted that this study has limitations: first, the method’s application is currently limited to meat-based pre-prepared dishes, and its adaptability to high-starch and high-fiber matrix samples remains unverified; second, the interference mechanisms of processing parameters (e.g., duration of high-temperature treatment, frequency of oil usage) on sterol detection results have not been systematically investigated. Future research will focus on two aspects: 1. Expanding the scope of sample matrices (e.g., plant-based and seasoned pre-prepared dishes) to validate method universality; 2. Establishing a correlation model linking “processing parameters—sterol changes—nutritional safety” by integrating sterol oxidation product detection, thereby further enhancing the method’s support for “safety-nutrition” control in pre-prepared dishes.

## Data Availability

The original contributions presented in the study are included in the article/supplementary material, further inquiries can be directed to the corresponding authors.
